# Evaluation of the recombinant protein *Sh*-TSP-2 for the serological diagnosis of imported urogenital schistosomiasis and comparison with commercially available tests

**DOI:** 10.1017/S0031182024001574

**Published:** 2025-01

**Authors:** María Pilar Luzón-García, Laura Navarro, Esther Rodríguez, Manuel Jesús Soriano-Pérez, José Vázquez-Villegas, María Isabel Cabeza-Barrera, Alex Loukas, Nerea Castillo-Fernández, María Jesús Perteguer, Javier Sotillo, Joaquín Salas-Coronas

**Affiliations:** 1 Tropical Medicine Unit, Hospital Universitario Poniente, Ctra. de Almerimar 31, 04700 El Ejido, Spain; 2 CIBERINFEC, ISCIII, Instituto de Salud Carlos III, Madrid, Spain; 3 Parasitology Reference and Research Laboratory, Centro Nacional de Microbiología, Instituto de Salud Carlos III, Madrid, Spain; 4 Tropical Medicine Unit, Distrito Poniente de Almería, Almería, Spain; 5 Australian Institute of Tropical Health and Medicine, James Cook University, Cairns, Australia; 6 Department of Nursing, Physiotherapy and Medicine, Faculty of Health Sciences, University of Almería, 04120 La Cañada, Spain

**Keywords:** diagnostics, migrants, *Schistosoma haematobium*, travellers, urogenital schistosomiasis

## Abstract

Different agencies have emphasized the need to evaluate current serological methods for screening patients with suspected urogenital schistosomiasis. However, there is still a lack of evidence regarding the most appropriate methods for this purpose. Here we assessed the diagnostic efficacy of a newly developed serological technique that utilizes the recombinant protein *Sh*-TSP-2, applied to the urine and serum of migrants suspected of having urogenital schistosomiasis. The sensitivity, specificity, positive and negative predictive values of an in-house enzyme-linked immunosorbent assay (ELISA) using the recombinant protein *Sh*-TSP-2 were analysed and compared with other commercial serological methods. Due to the limitations of microscopy as a perfect reference method, a latent class analysis (LCA) and composite reference standard (CRS) approach was used to determine the sensitivity and specificity of each test. According to the LCA model, the commercial tests NovaLisa^®^ and immunochromatography test (ICT) immunoglobulin G–immunoglobulin M (IgG–IgM) presented the highest sensitivity (100%), whereas the *Sh*-TSP-2 serum ELISA test had 79.2%. The *Sh*-TSP-2 urine and serum ELISA tests had the highest specificities among the serological methods (87.5 and 75%, respectively). CRS modelling showed that the ICT IgG–IgM, NovaLisa^®^ and *Sh*-TSP-2 serum tests led in sensitivity at 97.1, 88.6 and 71.4%, respectively, with all tests except that the ICT IgG–IgM test having a specificity >90%. *Sh*-TSP-2 has been validated as a screening tool for patients suspected of having urogenital schistosomiasis. Although commercial serological tests have shown higher sensitivities, *Sh*-TSP-2 could be valuable for confirming results from tests with lower specificity. Nevertheless, further studies with larger patient cohorts are necessary to fully verify its potential.

## Graphical abstract

**Figure:**
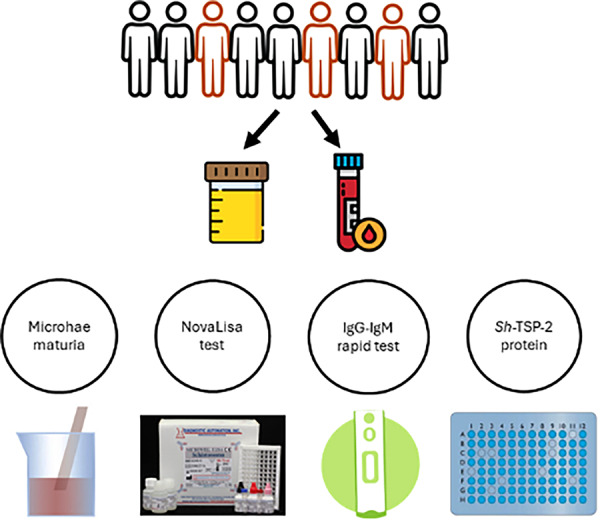

## Introduction

Schistosomiasis, caused by parasites of the genus *Schistosoma*, is a chronic and debilitating disease that affects more than 200 million people globally, imposing a substantial burden with 1.4 million disability-adjusted life years (GBD 2017 DALYs and HALE Collaborators, 2018). Among the 6 species that can infect humans, *Schistosoma haematobium* is responsible for more than half of all infections, resulting in urogenital schistosomiasis and a variety of health problems (Steinmann *et al*., 2006; McManus *et al*., 2018). Indeed, infection with *S. haematobium* has been associated with urinary disorders such as vesicoureteral reflux and hydronephrosis, as well as genital disorders such as ulcers and nodules in vulva, perineum and cervix in women and chronic prostatitis oligospermia and dyspareunia in men (McManus *et al*., 2018; Santos *et al*., 2021). More importantly, urogenital schistosomiasis has been associated with squamous cell carcinoma of the urinary bladder, and *S. haematobium* has been categorized by the World Health Organization (WHO) International Agency for Research on Cancer (IARC) as a group 1 carcinogen (Vennervald and Polman, 2009; IARC Working Group on the Evaluation of Carcinogenic Risks to Humans, 2012).

Although urogenital schistosomiasis is mainly localized in sub-Saharan Africa and the Middle East, it has been reported in up to 54 different countries (McManus *et al*., 2018), and, furthermore, autochthonous transmission has recently been described in 2 European countries (France and Spain) in the last few decades (Ramalli *et al*., 2018; Salas-Coronas *et al*., 2021). Despite these 2 reported outbreaks, the majority of diagnosed cases in Europe are a result of imported infections. A study published recently by the TropNet network found that half of the cases diagnosed in Europe occurred in migrants, whereas more than a third of the diagnoses occurred in European travellers and only 16% in long-term expatriates (Witzenrath *et al*., 2017). Although a consensus has recently been reached on the definition of imported schistosomiasis (Tamarozzi *et al*., 2024), the way of carrying out the diagnosis of the disease has been very variable, with serological techniques being used on many occasions. In these cases, the occurrence of schistosomiasis within the migrant population varies depending on the diagnostic technique employed, with higher rates observed when serological tests are used (Witzenrath *et al*., 2017). Furthermore, the same study found that only 27% of the infections diagnosed in travellers were acute and occurred in the first 3 months after contact, whereas most patients are diagnosed in chronic and advanced stages of the disease (Witzenrath *et al*., 2017). In the case of migrants, between 40 and 60% of patients with chronic schistosomiasis are asymptomatic, making the diagnostic delay even more evident (Whitty *et al*., 2000; Marchese *et al*., 2018), which can lead to complications such as renal failure (Roure *et al*., 2017). The high prevalence in migrants and travellers returning from endemic areas along with the significant morbidity linked to delayed diagnosis has led the European health authorities (ECDC) to recommend screening in this population (European Centre for Disease Prevention and Control, 2018).

Although the WHO recommends the microscopic examination of eggs in filtered urine as the reference diagnostic method for urogenital schistosomiasis, this technique has limited sensitivity and is less valuable in regions with low transmission or those outside endemic areas (Kosinski *et al*., 2011; Knopp *et al*., 2015). Furthermore, detection of microhaematuria is also performed in endemic areas, although it may not always go to zero after praziquantel treatment and can result from other medical conditions, so results need to be interpreted with caution and it may not be an appropriate test in non-endemic settings (Kosinski *et al*., 2011; Ochodo *et al*., 2015). Molecular tests, such as quantitative polymerase chain reaction (qPCR) amplifying the *S. haematobium* Dra1 repetitive motif and real-time fluorescence recombinase polymerase amplification targeting the same motif, as well as various genus- and species-specific loop-mediated isothermal amplification assays, have been developed, demonstrating high sensitivity and specificity (Cnops *et al*., 2013; Rostron *et al*., 2019; García-Bernalt Diego *et al*., 2021). However, they have not been implemented in most routine laboratories yet and are not suited to field implementation due to the requirement for scientific instruments to perform the assays. Antigenic tests, such as the circulating cathodic antigen in urine, also suffer from important limitations for the diagnosis of *Schistosoma* species other than *Schistosoma mansoni* and for the diagnosis of patients in non-endemic regions (Beltrame *et al*., 2017*a*), and circulating anodic antigen is not yet commercialized. Although serological tests cannot distinguish from current or past infections, they can be useful for screening purposes in non-endemic countries and can be easily implemented in routine laboratories and even in primary health care. Indeed, current recommendations favour use of serological methods for screening schistosomiasis in non-endemic regions such as Europe, Australasia and Canada (Pottie *et al*., 2011; Chaves *et al*., 2017; European Centre for Disease Prevention and Control, 2018).

The absence of suitable and validated tests in non-endemic regions has resulted in significant variability in patient management across different healthcare centres. Indeed, national guidelines for the follow-up of these patients and the handling of potential complications are currently lacking, and only recently, consensus documents have been published in Spain and Italy among other countries to serve as a guide for the screening, diagnosis and treatment of this disease outside endemic areas (Bocanegra *et al*., 2023; Comelli *et al*., 2023). The primary aim of this study was to validate a recently developed enzyme-linked immunosorbent assay (ELISA) test, which utilizes the recombinant protein *Sh*-TSP-2 (Pearson *et al*., 2021), for the diagnosis and screening of *Schistosoma* infections in migrant populations using both serum and urine samples. Additionally, we aimed to compare the performance of this ELISA with existing standard serological commercial kits such as the *Schistosoma* immunochromatography technology (ICT) immunoglobulin G–immunoglobulin M (IgG–IgM) lateral flow immunochromatography test and the NovaLisa^®^ *S. mansoni* IgG ELISA. This study provides important information on the standardization of adequate screening methods against urogenital schistosomiasis and the management of this important infectious disease.

## Materials and methods

### Study design and study population

A prospective observational study comparing diagnostic tests for the detection and screening of schistosomiasis was carried out in sub-Saharan migrant patients attending the Tropical Medicine Unit (TMU) of the Hospital Universitario Poniente (El Ejido, Almería, Spain) from January 2020 to June 2021. Patients included were sub-Saharan migrants older than 18 years of age. Patients with HIV infection were excluded. Furthermore, the serum and urine of 8 and 10 patients, respectively, were used as negative controls in the in-house serological ELISA tests.

### Definitions and collected data

A screening protocol for infectious diseases was systematically applied to all migrant patients referred to the TMU. For sub-Saharan migrant patients, the screening protocol comprised several tests: blood count, liver and renal function tests, syphilis, HIV, hepatitis B virus and hepatitis C virus serologies, tuberculin skin test and search for parasites in stool (3 concentrated stool samples) and urine (1 concentrated urine sample [10 cm^3^]), *Strongyloides* (ELISA DRG^®^ Strongyloides IgG) and Knott and/or saponin tests for microfilariae as well as chest and abdominal X-rays. If any other specific disease was suspected (e.g. onchocerciasis, malaria, etc.), further diagnostic procedures were performed. Diagnosis of strongyloidiasis was considered positive either when larvae were isolated from stool samples or when serology was positive. Patients positive to these diseases were excluded from the study.

Diagnosis of schistosomiasis was confirmed when *Schistosoma* spp. eggs were microscopically detected in urine and/or feces. Patients with confirmed schistosomiasis and those with a *Schistosoma* positive serology result were treated with praziquantel at the usual doses (40 mg/kg, 1 day), completing the study with abdominal and bladder ultrasound in cases of confirmed schistosomiasis or when they presented genitourinary symptoms.

### Microscopy

Urine samples were collected between 9 a.m. and 12 p.m. and processed on the day of the sample collection. After centrifugation at 1500 rpm for concentration, each urine sample was placed on a labelled slide and examined under a microscope (100×) for *Schistosoma* eggs. Aliquots of urine samples were reserved and stored at −80°C until shipment to a laboratory at Centro Nacional de Microbiología (Madrid, Spain) for further serological analyses.

Additionally, to discard the possibility of *S. mansoni* infections and co-infections, 3 stool samples per patient were collected in formol on alternate days. Each sample was subjected to formol–ether concentration (Ritchie's method) and 3 slides were examined microscopically (100×).

### IgG–IgM rapid test

The *Schistosoma* ICT IgG–IgM lateral flow immunochromatography test (LDBIO Diagnostics, Lyon, France) was used for the detection of IgG and IgM antibodies against *S. haematobium* in the serum of patients following the manufacturer's instructions. Briefly, 30 μL of serum were deposited in the sample well, followed by 3 drops of the supplied eluent. The result was considered positive when a band was visible in the corresponding test and control areas, whereas the results were considered negative when only a blue control band was visible, or in the absence of test line.

### Recombinant protein production

The recombinant protein *Sh*-TSP-2 was expressed and purified as described previously (Pearson *et al*., 2021). In brief, 10 mL of Luria broth containing 50 μg mL^−1^ kanamycin (LB_kan_) was inoculated with *Escherichia coli* BL21 (DE3) containing the protein-encoding plasmid and incubated overnight at 37°C with shaking at 200 rpm. Overnight culture was seeded (1/100) into 500 mL of fresh LB_kan_ and incubated at 37°C with shaking at 200 rpm until optical density (OD_600_) = 0.5–1.0 (approximately 3 h). Protein expression was induced for 24 h by addition of 1 mm isopropyl beta-d-1-thiogalactopyranoside using standard methods. Cells were harvested by centrifugation (8000 ***g*** for 20 min at 4°C), resuspended in 50 mL of lysis buffer (50 mm sodium phosphate at pH 8, 40 mm imidazole and 300 mm NaCl), and subjected to 3 cycles of freeze/thawing followed by sonication (10× 5 s pulses [70% amplitude] with 30 s rest periods between each pulse) with a FB50 sonic dismembrator (Fisher Scientific, Hampton, USA) at 4°C. The bacterial lysate was finally centrifuged at 20 000 ***g*** for 20 min at 4°C and the supernatant decanted and stored at −80°C.

An ÄKTA Pure™ UPC FPLC (GE Healthcare, Chicago, USA) was used to purify the recombinant proteins using Ni^2+^ immobilized metal ion affinity chromatography (IMAC). The recombinant protein solutions were diluted at a ratio of 1:4 in lysis buffer and subsequently passed through a 0.45 μm filter. These solutions were introduced into a 1 mL His-Trap IMAC column (GE Healthcare, USA) that had been pre-equilibrated with lysis buffer at a flow rate of 1 mL min^−1^. Bound proteins were washed with 10 column volumes of lysis buffer and then eluted using lysis buffer with an increasing linear gradient of imidazole (100–500 mm). Fractions containing the purified recombinant protein with the highest purity were pooled and subjected to buffer exchange into phosphate-buffered saline (PBS) using an Amicon Ultra-15 centrifugal filter with a molecular weight cutoff of 3 kDa. The identity of expressed proteins was confirmed by SDS-PAGE and western blot using anti-His monoclonal antibodies (Invitrogen, Massachusetts, USA).

### ELISA assays

For the detection of antibodies against *S. haematobium* in the urine or serum of patients, 2 different ELISAs were performed. The commercial kit NovaLisa^®^ *S. mansoni* IgG ELISA (Gold Standard Diagnostics Frankfurt GmbH, Dietzenbach, Germany) was performed following the manufacturer's instructions using a DS2 Automated ELISA System (Dynex). Briefly, 100 μL of control and serum samples (diluted 1:100) were added to the pre-coated plates and incubated at 37°C for 1 h. After washing with 300 μL of a washing solution, 100 μL of peroxidase-labelled protein A was added to each well and plates were incubated for 30 min at room temperature (RT). Finally, plates were washed with a washing solution and developed using 100 μL of 3,3′,5,5′-tetramethylbenzidine (TMB) for 15 min, followed by 100 μL of stop solution. Plates were read at a wavelength of 492 nm on a Multiskan FC (Thermo Fisher, Massachusetts, USA) microplate reader.

For the in-house *Sh*-TSP-2 ELISAs Nunc™ MicroWell™ 96-Well Medisorp™ Microplates (Thermo Fisher) were coated overnight at 4°C with 2 μg mL^−1^ of *Sh*-TSP-2 in 0.1 m Na_2_CO_3_/NaHCO_3_ at pH 9.6. The following day the plates were washed with PBS-Tween-20 at 0.05% (PBST) and blocked for 2 h at RT with 100 μL of either PBST/5% skimmed milk powder (for urine samples) or PBST/5% bovine serum albumin (BSA) (for serum samples). Fifty microlitres of urine (1:10 in PBST) or serum (1:500 in PBST/1% BSA) were added to the wells and incubated overnight at 4°C. The following day the plates were washed with PBST and incubated for 1 h at RT with 100 μL of goat anti-human IgG-HRP (Sigma-Aldrich, St. Louis, USA, 1:5000 in PBST). Finally, after washing with PBST, TMB was added to the plates and the oxidation reaction was stopped with 0.5 m sulphuric acid and read at a wavelength of 492 nm on a Multiskan FC (Thermo Fisher) microplate reader.

### Reference standard calculation

Due to the limitations of microscopy as a perfect reference method, 2 different approaches were followed to calculate the accuracy of the tests performed. First, a latent class analysis (LCA) was carried out using the randomLCA R package (Beath, 2017) to determine the sensitivity and specificity of each of the tests employed. This analysis combines the results of multiple diagnostic tests through a probabilistic model to obtain estimates of disease prevalence and diagnostic test accuracy in situations where there is no single, accurate reference standard. In this case, the confirmed *S. haematobium* infection status was considered as a latent variable with 2 categories: ‘infected’ and ‘non-infected’. Sensitivity (probability that a test result will be positive when the disease is present) and specificity (probability that a test result will be negative when the disease is not present) were estimated for each test by relating the true disease class and the observed test results.

Second, a composite reference standard (CRS) was calculated using the previously published methods (Reitsma *et al*., 2009; Beltrame *et al*., 2017*a*). In this case, patients were classified as infected if they presented a positive microscopy result OR a negative microscopy result AND at least 2 concordant positives of the 3 index tests. Contrarily, patients were classified as non-infected when they had a negative microscopy result AND less than 2 positives of the 3 index tests. Index tests included the following: *Schistosoma* ICT IgG–IgM lateral flow immunochromatography test (LDBIO Diagnostics), NovaLisa^®^ *S. mansoni* IgG ELISA and the *Sh*-TSP-2 in-house test (performed in urine and/or serum).

### Statistical analysis

In-house *Sh*-TSP-2 ELISA was carried out in triplicate and visualized using GraphPad Prism 7. Reactivity cutoffs were determined as the average plus 3 s.d. of the values of the non-endemic negative group. Receiver operating characteristic (ROC) curves were constructed using GraphPad Prism 7. Prior to the statistical analyses, normal distribution of the data was assessed using GraphPad Prism 7. To assess statistical significance, the non-parametric Mann–Whitney test was employed to compare the eosinophilia values between the groups. The parametric Student's *t*-test was employed to compare the mean antibody responses of each infected group to those of the non-infected group, with the significance levels presented as follows: *P ⩽ 0.05, **P ⩽ 0.005, ***P ⩽ 0.0005. Furthermore, the relationship between the intensity of infection determined by microscopy and eosinophilia was examined by the Spearman correlation test. This test measures the strength and direction of a monotonic relationship between 2 variables even when data are not normally distributed. A *P* value lower than 0.05 was considered statistically significant.

## Results

### Study participants and biochemical analyses

A total of 60 participants were enrolled in the study; however, 4 participants were removed from further analyses since they tested positive to *S. mansoni* but not to *S. haematobium* by microscopy. The matching urine and serum samples from the remaining 56 participants were collected and analysed. Out of the 56 patients included, 51 (91.1%) were men, had a mean age of 27.8 years (s.d. 7.05) and a mean stay in Spain of 21.02 months (s.d. 27.36). All participants came from West African countries, mainly Mali (*n* = 22; 39.3%), Senegal (*n* = 18; 32.11%) and Mauritania (*n* = 5, 8.9%). In total, 89% were referred from primary care. The main reasons for consultation were macroscopic haematuria (*n* = 20, 35.7%) and abdominal pain (*n* = 6, 10.5%). In total, 31 patients (55%) presented eosinophilia (>450 Eo mm^−3^), with a mean of 453.9 Eo mm^−3^ (s.d. 459.7). In relation to other co-parasites, 1 patient was diagnosed with strongyloidiasis, 1 with *Plasmodium falciparum* malaria, as well as intestinal protozoa (32 *Blastocystis hominis*, 3 *Entamoeba histolytica*/*dispar* and 2 *Giardia lamblia*).

**Figure 1.**
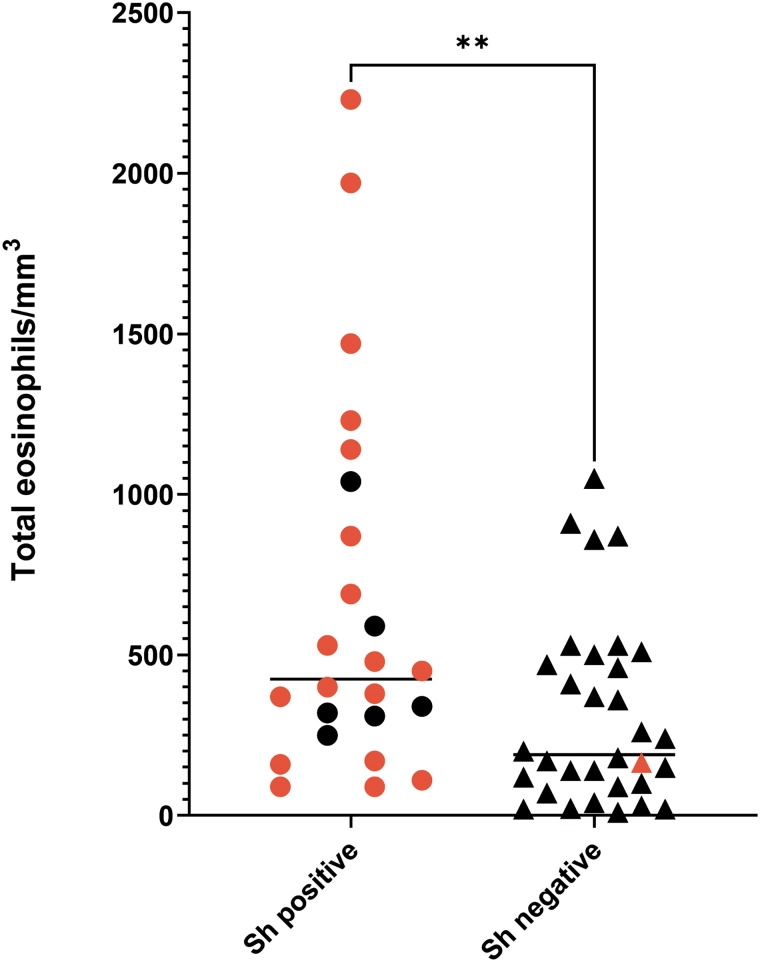
Eosinophilia values in *Schistosoma haematobium*-infected and -uninfected participants. Red dots/triangles correspond to participants also showing microhaematuria. **P ⩽ 0.005.

According to the microscopy analysis, 42.86% of the participants (24/56) were positive for *S. haematobium* infection, with egg numbers varying from 0.1 to 56 eggs/10 mL, and a median of 6.9 (95% confidence interval [CI] [2.8, 10.5]). Of the patients who were positive by microscopy, 18 presented with microhaematuria. Only 1 egg-negative participant had microhaematuria. Furthermore, eosinophilia was found in 10 *S. haematobium*-positive patients (41.7%), whereas 8 non-infected participants showed elevated eosinophilia (25%) (Fig. 1). No statistically significant correlation between intensity of infection (number of eggs) and the eosinophilia values was observed for the *S. haematobium*-infected group (*P* = 0.796; *r* = 0.055).

### Suitability of the *Sh*-TSP-2 test for the diagnosis of urogenital schistosomiasis in migrants

**Figure 2.**
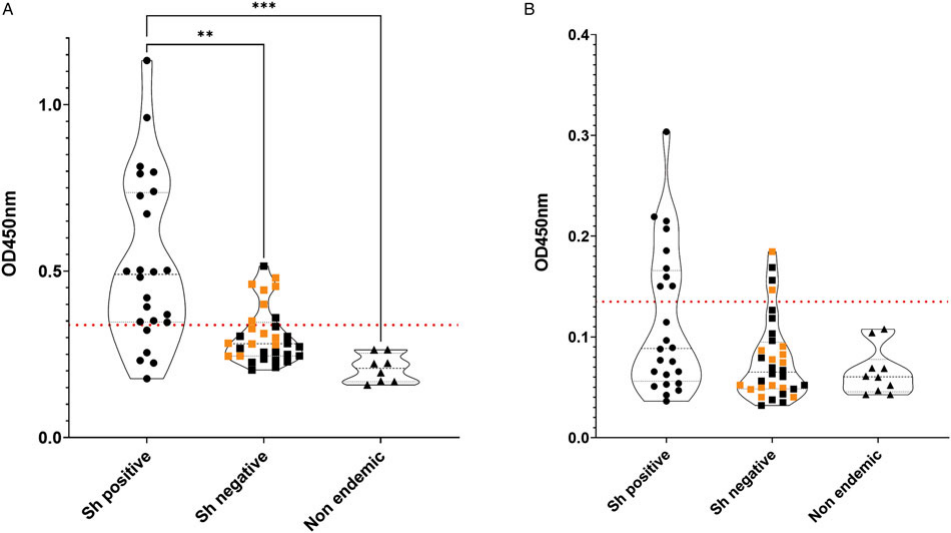
IgG antibody responses to *Sh*-TSP-2 in serum (A) and urine (B) of screened patients. Orange squares from the *S. haematobium* negative group (negative by microscopy) correspond to patients shown positive to the *Schistosoma* ICT IgG–IgM test. Horizontal red dotted line represents test cutoff. **P ⩽ 0.005, ***P ⩽ 0.0005.

All 56 participants were tested with the *Sh*-TSP-2 test, and the urine and serum from non-endemic volunteers (8 and 10 patients, respectively) with no travel history to endemic areas of schistosomiasis were used to determine the cutoff of the test (0.331 and 0.136 for serum and urine samples, respectively) (Fig. 2).

The serum from 79.1% of the samples positive by microscopy (19 out of 24) were significantly reactive to *Sh*-TSP-2, whereas only 8 out of 32 (25.8%) of the samples negative by microscopy were positive by ELISA. Interestingly, from these negative by microscopy samples, 75% of the positive by ELISA (6 out of 8) were also positive by the ICT IgG–IgM test (Fig. 2A, orange squares), and 8 out of 24 ELISA negative samples (33.3%) were positive by the ICT IgG–IgM test. In addition, the OD values from the *S. haematobium* infected group were, in general, significantly higher than the non-infected group (*P* ⩽ 0.005) and the control group (*P* ⩽ 0.0005). Furthermore, the area under the curves (AUC) generated from the ROC curves were used to determine the sensitivity and specificity as described previously (Pearson *et al*., 2021) (Supplementary Fig. 1). In the case of serum, the AUC values from the ROC curves were 0.94 (Supplementary Fig. 1A).

In the case of urine, *Sh-*TSP-2 could only detect 9 out of 24 (37.5%) of the positive infections (Fig. 2B), and the OD values of the different population groups were not significantly different (Fig. 2B). Furthermore, the AUC values from the ROC curves were 0.71 (Supplementary Fig. 1B).

### Prevalence and intensity of *S. haematobium* infection according to the LCA and the CRS

**Figure 3.**
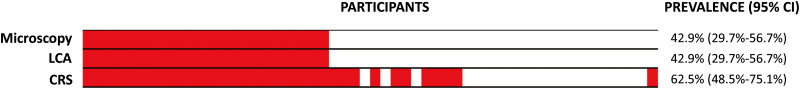
Frequency of recognition patterns for all individuals based on the different reference standards: microscopy, LCA and CRS.

First, an LCA was performed. In this case, the probability of each individual being classified as ‘infected’ based on the 6 tests performed was estimated from the fit of the latent class model, that included a random effect. The best model was chosen based on the lowest value of the Bayesian information criterion (BIC), and the posterior class probabilities for each observed pattern and class were determined. The LCA models with 2 classes had a smaller BIC statistic compared to models with either 1 or 3 classes, therefore the 2-class LCA model in random mode was chosen. Interestingly, the LCA did not affect the status of any individual, with all participants positive by microscopy being positive by LCA and all negative participants remaining as negatives by LCA. Based on this, the disease prevalence was 42.86% (95% CI [29.71, 56.78]) (Fig. 3).

Furthermore, the proportion of positive results according to the CRS was 62.5% (95% CI [48.55, 75.08]), with 35 participants presenting a positive microscopy result or a negative microscopy result and at least 2 concordant positives of the 3 index tests (Fig. 3).

### Sensitivities and specificities of the diagnostic assays

According to LCA modelling, microscopy as well as the commercial tests NovaLisa^®^ and the ICT IgG–IgM had the highest sensitivity (100%), followed by the *Sh-*TSP-2 in-house serum ELISA test (79.2%) (Table 1). However, the highest specificities, corresponded to the microscopy (100%), the microhaematuria test (96.9%) and the *Sh-*TSP-2 in-house urine and serum ELISA tests (87.5 and 75%, respectively) (Table 1). The detection of eggs by microscopy also had the highest values in terms of positive predictive value (PPV), negative predictive value (NPV) and accuracy, which was not surprising after the correlative results between microscopy and LCA modelling. The NovaLisa^®^ and the *Sh-*TSP-2 serum test showed similar PPV results with 72.7 and 70.4%, respectively. All tests had an accuracy ⩾75% except for the *Sh-*TSP-2 urine test (66.1%) (Table 1).

**Table 1.** Estimated parameters for the 6 different diagnostic tests according to LCA

	Sn (95% CI)	Sp (95% CI)	PPV (95% CI)	NPV (95% CI)	Accuracy (95% CI)	PLR	NLR
*Sh-*TSP2_serum	79.2% (57.9–92.9)	75% (56.6–88.5)	70.4% (55.7–81.7)	82.8% (68.2–91.5)	76.8% (63.6–87)	3.17 (1.7–6)	0.28 (0.1–0.6)
*Sh-*TSP2_urine	37.5% (18.8–59.4)	87.5% (71–96.5)	69.2% (44–86.6)	65.1% (57.1–72.3)	66.1% (52.2–78.2)	3.00 (1–8.6)	0.71 (0.5–1)
Microscopy	100% (85.7–100)	100% (89.1–100)	100% (85.7–100)	100% (89.1–100)	100% (93.6–100)	>99	0
Haematuria	75% (53.3–90.2)	96.9% (83.8–99.92)	94.7% (72–99.2)	83.8% (72–91.2)	87.5% (75.9–94.8)	24 (3.4–167.5)	0.26 (0.3–0.5)
NovaLisa^®^	100% (85.7–100)	71.9% (53.2–86.2)	72.7% (60.5–82.3)	100% (85.2–100)	83.9% (71.7–92.4)	3.56 (2–6.2)	0
ICT IgG_IgM	100% (85.7–100)	56.2% (37.7–73.6)	63.2% (53.6–71.7)	100% (81.5–100)	75% (61.6–85.6)	2.29 (1.5–3.4)	0

Sn, sensitivity; Sp, specificity; PPV, positive predictive value; NPV, negative predictive value; PLR, positive likelihood ratio; NLR, negative likelihood ratio; CI, confidence interval.

In the case of CRS modelling, the top 3 diagnostic tests in terms of sensitivity were the ICT IgG–IgM test (97.1%), the NovaLisa^®^ test (88.6%) and the *Sh-*TSP-2 serum test (71.4%) (Table 2). Only the microscopy test was performed with a specificity of 100%, whereas all the other tests had a specificity >90% except for the ICT IgG–IgM test (80.9%). Similarly, all tests had a PPV >90% except for the ICT IgG–IgM test (89.5%). The highest accuracy values corresponded to the ICT IgG–IgM test, the NovaLisa^®^ test, microscopy and the *Sh-*TSP-2 serum test (91.1, 89.3, 80.4 and 78.6%, respectively).

**Table 2.** Estimated parameters for the 6 different diagnostic tests according to CRS

	Sn (95% CI)	Sp (95% CI)	PPV (95% CI)	NPV (95% CI)	Accuracy (95% CI)	PLR	NLR
*Sh-*TSP2_serum	71.4% (53.7–85.4)	90.4% (69.6–98.8)	92.6% (76.7–97)	65.5% (52.50–76.6)	78.6% (65.6–88.4)	7.50 (2–28.5)	0.32 (0.2–0.5)
*Sh-*TSP2_urine	34.3% (19.1–52.2)	95.2% (76.2–99.9)	92.3% (62.7–98.8)	46.5% (40.2–52.9)	57.1% (43.2–70.3)	7.0 (1–51.5)	0.69 (0.5–0.9)
Microscopy	68.6% (50.7–83.1)	100% (83.9–100)	100% (85.7–100)	65.6% (53.9–75.7)	80.4% (67.6–89.8)	>99	0.31 (0.2–0.5)
Haematuria	51.4% (34–68.6)	95.2% (76.2–99.9)	94.7% (72.1–99.2)	54.1% (45.2–62.6)	67.9% (54–79.7)	10.80 (1.5–75.1)	0.51 (0.4–0.7)
NovaLisa^®^	88.6% (73.3–96.8)	90.5% (69.6–98.8)	93.9% (80.5–98.3)	82.6% (65.1–92.3)	89.3% (78.1–96)	9.30 (2.5–34.9)	0.13 (0.1–0.3)
ICT IgG_IgM	97.1% (85.1–99.9)	80.9% (58.1–94.5)	89.5% (77.8–95.4)	94.4% (70.9–99.2)	91.1% (80.4–97)	5.10 (2.1–12.3)	0.04 (0–0.2)

Sn, sensitivity; Sp, specificity; PPV, positive predictive value; NPV, negative predictive value; PLR, positive likelihood ratio; NLR, negative likelihood ratio; CI, confidence intervals.

### Concordance of the subject classification between CRS and LCA models

Subjects with at least 2 concordant positive results of the index tests OR with a positive microscopy (irrespective of the other results) had 68.5% probability of being classified as cases by LCA, whereas all the others (negative microscopy and <2 positive index tests) had 100% probability of being classified as non-cases by LCA. The concordance between the 2 methods of classification was substantial (Cohen's kappa = 0.62).

### Predictive values of a combination of 2 tests

As the ICT IgG–IgM test had the greatest sensitivity in both CRS and LCA models, but not the highest specificity, we assessed the PPV and NPV of a combination of a positive ICT IgG–IgM test with a positive or a negative second test, respectively. The PPV and NPV values estimate the likelihood that an individual truly has the disease given a positive test result (PPV) or does not have the disease given a negative test result (NPV). In this case, when LCA was used as the reference standard model, the highest PPV was observed when combining the ICT IgG–IgM test with the *Sh-*TSP-2 serum test (65.5%, Table 3). Furthermore, the combination with this test displayed a high NPV (81.5%), although lower than the combination with microscopy and NovaLisa^®^ (both having 100%) (Table 3). In terms of accuracy, the highest accuracy was observed when combining the ICT IgG–IgM test and microscopy (75%), followed by the combination ICT IgG–IgM and *Sh-*TSP-2 serum tests (73.21%) (Table 3).

**Table 3.** Estimated PPV, NPV and accuracy of the test for the combination of positive ICT IgG–IgM test and a positive or negative second test in both models

	LCA			CRS		
Second test	PPV (95% CI)	NPV (95% CI)	Accuracy (95% CI)	PPV (95% CI)	NPV (95% CI)	Accuracy (95% CI)
*Sh-*TSP-2_serum	65.5% (52.2–76.8)	81.5% (66.1–90.8)	73.2% (59.7–84.2)	80.6% (67.2–89.4)	60% (45.4–73)	71.4% (57.8–82.7)
*Sh-*TSP-2_urine	36% (23.2–51.2)	51.6% (40.1–62.9)	44.6% (31.3–58.5)	68.7% (47–84.5)	40% (32.4–48.1)	48.2% (34.7–62)
Microscopy	63.2% (53.6–71.7)	100% (81.5–100)	75% (61.6–85.6)	85.7% (70.7–93.7)	60.7% (47.6–72.4)	73.2% (59.7–84.2)
Haematuria	54.5% (43.7–65)	73.9% (56.8–85.9)	62.5% (48.5–75.1)	78.3% (61.1–89.2)	48.5% (38.3–58.8)	60.7% (46.7–73.5)
NovaLisa^®^	58.5% (50.5–66.1)	100% (78.2–100)	69.6% (55.9–81.2)	66.7% (59.6–73)	54.5% (29.4–77.5)	64.3% (50.4–76.6)

PPV, positive predictive value; NPV, negative predictive value; CI, confidence intervals.

In the case of CRS as the reference standard model, the combination of the ICT IgG–IgM test with the *Sh-*TSP-2 serum test had the second highest PPV (80.65%), as well as the second highest NPV (60%) and accuracy (71.43%) only after the combination of the ICT IgG–IgM test with microscopy (Table 3).

## Discussion

Urogenital schistosomiasis is mainly localized and endemic in sub-Saharan Africa and several countries in the Middle East (McManus *et al*., 2018); however, recent studies have reported endemic transmission of *S. haematobium* in European regions such as Corsica (France) and Almeria (Spain) (Rothe *et al*., 2021; Salas-Coronas *et al*., 2021). It has been previously shown that between 10 and 20% of sub-Saharan immigrants are positive to *S. haematobium* by microscopy, but this prevalence could be higher due to the low sensitivity of this diagnostic method (Beltrame *et al*., 2017*b*; Serre-Delcor *et al*., 2017; Asundi *et al*., 2019; Roade *et al*., 2023). Of these, up to 40–60% are asymptomatic, highlighting the importance of post-travel consultations and screening of patients from endemic regions or long-term visitors to reduce the number of missed diagnoses (Whitty *et al*., 2000; Witzenrath *et al*., 2017).

Serology is a highly sensitive method for diagnosing schistosomiasis, particularly in cases of low-intensity infections. It is primarily used in epidemiological research, control efforts and in the field of travel medicine (reviewed by Hinz *et al*., 2017). Due to the recommendation of using serological methods for the screening of migrants by ECDC and several national societies, in the current study, we aimed to validate the recombinant protein *Sh*-TSP-2 for the diagnosis and screening of *Schistosoma* infections in migrant populations using serum and urine samples by ELISA. Furthermore, we sought to compare the performance of this technique with the established commercial serological tests, including the *Schistosoma* ICT IgG–IgM lateral flow immunochromatography test and the NovaLisa^®^ *S. mansoni* IgG ELISA.

The *Sh*-TSP-2 test demonstrated sensitivities similar to those previously reported in endemic areas (Pearson *et al*., 2021), with a sensitivity of 79.2% using serum samples when LCA was used as the reference standard. However, when CRS was the reference standard, the sensitivity was slightly lower at 71.4%. Despite the lower sensitivity compared to commercial methods, *Sh*-TSP-2 presented a high specificity, particularly when using urine, which can be important for patients from areas where other helminths are also endemic. It is worth noting that LCA calculates 100% sensitivity for microscopy. In this regard, the relatively low number of samples and the fact that most indicators were serological tests, could have influenced the statistical power of the LCA.

Although there is no consensus on which patients should be screened, generally, high levels of blood eosinophils are usually indicative of a potential helminth infection. To evaluate the potential correlation of eosinophilia and *S. haematobium* infection in our cohort, we first analysed this biochemical parameter; however, in our study, not all patients with confirmed *S. haematobium* infections presented eosinophilia. Although eosinophilia is usually associated with *Schistosoma* and other helminth infections, it has been documented that less than 50% of patients with chronic schistosomiasis had associated eosinophilia (Tamarozzi *et al*., 2021), and, thus, this parameter is not sufficient for the diagnosis and screening of patients. Furthermore, 75% of patients with confirmed urogenital schistosomiasis presented microhaematuria, whereas 6 did not, and only 1 of the negative patients showed microhaematuria. Although haematuria is not specific to *S. haematobium* infections, and could be a symptom of other genitourinary infections, due to the low cost and simplicity of this test, it could be useful as a first step in the screening of urogenital schistosomiasis.

Our results indicate that the ICT IgG–IgM test showed the highest sensitivity when diagnosing imported urogenital schistosomiasis, independently of the reference standard used. This is in accordance with the previous results showing the suitability of the ICT IgG–IgM test for the diagnosis of *S. mansoni* and *S. haematobium* infections (Beltrame *et al*., 2017*a*). The ability of this test to detect IgM and, thus, allow for an earlier identification of cases, could also be relevant in the diagnosis of acute schistosomiasis and returning travellers with Katayama fever, although more studies should be performed in this regard. When considering CRS as the reference standard, we also obtained similar results regarding specificity and NPV for this test. Indeed, this technique has been proposed as the best technique to screen at-risk individuals in clinical practice (Leblanc *et al*., 2021; Hoermann *et al*., 2022; Luzón-García *et al*., 2023). In the case of the NovaLisa^®^, our results showed a high sensitivity and specificity, which does not align with the previously published results. Kinkel *et al*. found a sensitivity of 35.7% (14.0–64.4) for this test in *S. haematobium*-infected patients and Luzón *et al*. showed a lower sensitivity in the case of *S. haematobium* infections (65.7%) compared to *S. mansoni* infections (82.1%) (Kinkel *et al*., 2012; Luzón-García *et al*., 2023). The high variability of sensitivities in different studies has been attributed to seroconversion (Kinkel *et al*., 2012). Although most individuals will have detectable IgG antibodies within 3 months after infection (antibodies typically begin to develop between 4 and 7 weeks post-infection), some may remain seronegative for as long as 6 months (Jones *et al*., 1992; Golledge, 1995). All our patients were migrants originating from sub-Saharan African countries, and, thus, positive patients most likely presented with chronic schistosomiasis (positive seroconversion). Furthermore, it has been previously suggested that detecting infections from hybrid forms might present challenges for serological diagnosis. None of the above-mentioned studies have confirmed the genotype of *Schistosoma* specimens (Hinz *et al*., 2017), and neither have we, so we cannot rule out this possibility. More studies in this regard should be performed.

It is well known that, in the case of schistosomiasis, serology remains positive for prolonged periods, particularly in migrants, and can still be positive even 3 years after treatment (Yong *et al*., 2010). Although this is true with commercial tests, where crude antigens are used, some studies have highlighted the decline of IgG levels against recombinant proteins as soon as 2 weeks after treatment (Mohammed *et al*., 2020). In this sense, the use of *Sh*-TSP-2 for monitoring treatment success should be further validated to determine antibody decay rates. Interestingly, when using urine as the sample, sensitivity dropped to 34–35% independently of the reference standard used. Repeated exposure to *Schistosoma* causes increased genitourinary problems, potentially leading to a greater leakage of antibodies from the serum into the urine (Mekonnen *et al*., 2022). Given the low infection rates observed in our study, coupled with the longer period since migrants' last exposure to *Schistosoma*, the likelihood of antibody leakage is reduced, suggesting that urine may not be a reliable sample for detecting antibodies in these cases, and possibly explaining the lower sensitivity reported here compared to that reported by Pearson *et al*. from *S. haematobium* endemic areas in Africa (Pearson *et al*., 2021).

Because of the antigens used in their preparations, mainly crude worm and egg preparations, current serological tests offer high sensitivity at the expense of sub-optimal specificity (Kinkel *et al*., 2012; Mesquita *et al*., 2022). Despite the need for a highly specific test in endemic settings, in non-endemic scenarios sensitivity is the priority, and a disjunctive approach using 2 or more serological tests where a positive result in any one of these would be considered indicative of schistosomiasis, has been proposed (Kinkel *et al*., 2012). In our case, due to the already high sensitivity observed for the ICT IgG–IgM test we do not see any potential benefit for the combination of 2 or more tests. Furthermore, the combination of serological and nucleic acid amplification tests (NAATs) has been proposed for the diagnosis of *Schistosomiasis mansoni*, increasing the accuracy of the diagnostic test from 71 to 94% (Mesquita *et al*., 2022). In our case, due to sample limitation, a qPCR or other NAATs could not be performed, although future prospective analyses in this sense will be performed. In this regard, to our knowledge, no studies have been performed analysing the combination of serological and NAATs for the diagnosis of urogenital schistosomiasis specifically.

In conclusion, we have validated the potential of *Sh*-TSP-2 for screening patients with suspected urogenital schistosomiasis, either independently or in conjunction with other serological techniques. However, other commercial serological tests, particularly the ICT IgG–IgM test, have shown better sensitivities. Nevertheless, further studies with larger cohorts are needed to confirm this potential. Furthermore, as an ELISA test, *Sh*-TSP-2 has some usability limitations (e.g. trained personnel, laboratory equipment needed) compared to a rapid test such as the ICT IgG–IGM, although its higher specificity could be an advantage in certain situations.

The present study has inherent limitations. First, only 1 urine sample from each patient was analysed by microscopy. Given the known daily fluctuation of *S. haematobium* oviposition, 3 different samples from alternate days should have been analysed to provide a true negative result as recommended by the CDC. Second, although patients were selected merely by positivity and negativity to microscopy analysis, not by symptomatology and infection intensity, and samples were sent blinded for the serological analysis, some selection bias cannot be ruled out. Third, despite the analysis of matching serum and urine samples, the number of patients screened was small and patients with other *Schistosoma* infections (i.e. *S. mansoni*) were excluded from the analysis, which could have shed light on the specificity of each technique. In this sense, the low number of samples could have affected the statistical power of the LCA. On the contrary, our study has several strengths, including the use of the alternative reference standards CRS and LCA. In this regard, evaluating the suitability of alternative diagnostic tests in comparison to a gold standard with poor sensitivity (which is the case of microscopy) is not ideal, and the use of alternative methods such as these is recommended (Reitsma *et al*., 2009). Although it is true that including the test being evaluated as part of the benchmark used to assess its performance could introduce bias when performing an analysis using CRS as the standard method, it is noteworthy that other studies have also employed this approach to estimate the accuracy of most available tests (Beltrame *et al*., 2017*a*).

## Supporting information

Luzón-García et al. supplementary material Luzón-García et al. supplementary material

## Data Availability

The datasets used and/or analysed during the current study are available from the corresponding author on reasonable request.

## Supplementary material

The supplementary material for this article can be found at https://doi.org/10.1017/S0031182024001574
